# Probiotic supplementation affects markers of intestinal barrier, oxidation, and inflammation in trained men; a randomized, double-blinded, placebo-controlled trial

**DOI:** 10.1186/1550-2783-9-45

**Published:** 2012-09-20

**Authors:** Manfred Lamprecht, Simon Bogner, Gert Schippinger, Kurt Steinbauer, Florian Fankhauser, Seth Hallstroem, Burkhard Schuetz, Joachim F Greilberger

**Affiliations:** 1 Institute of Physiological Chemistry, Centre for Physiological Medicine, Medical University of Graz, Graz, Austria; 2 Institute of Nutrient Research and Sport Nutrition, Graz, Austria; 3 SportchirurgiePlus, Centre for Individual Sport Medicine and Surgery, Graz, Austria; 4 Biovis Diagnostik MVZ GmbH, Limburg, Germany; 5 Institute of Laboratory Sciences, Dr Greilberger GmbH, Laßnitzhöhe, Austria

**Keywords:** Probiotics, Leaky gut, Intense exercise, Oxidative stress and inflammation

## Abstract

**Background:**

Probiotics are an upcoming group of nutraceuticals claiming positive effects on athlete’s gut health, redox biology and immunity but there is lack of evidence to support these statements.

**Methods:**

We conducted a randomized, double-blinded, placebo controlled trial to observe effects of probiotic supplementation on markers of intestinal barrier, oxidation and inflammation, at rest and after intense exercise. 23 trained men received multi-species probiotics (10^10^ CFU/day, Ecologic®Performance or OMNi-BiOTiC®POWER, n = 11) or placebo (n = 12) for 14 weeks and performed an intense cycle ergometry over 90 minutes at baseline and after 14 weeks. Zonulin and α1-antitrypsin were measured from feces to estimate gut leakage at baseline and at the end of treatment. Venous blood was collected at baseline and after 14 weeks, before and immediately post exercise, to determine carbonyl proteins (CP), malondialdehyde (MDA), total oxidation status of lipids (TOS), tumor necrosis factor-alpha (TNF-α), and interleukin-6 (IL-6). Statistical analysis used multifactorial analysis of variance (ANOVA). Level of significance was set at p < 0.05, a trend at p < 0.1.

**Results:**

Zonulin decreased with supplementation from values slightly above normal into normal ranges (<30 ng/ml) and was significantly lower after 14 weeks with probiotics compared to placebo (p = 0.019). We observed no influence on α1-antitrypsin (p > 0.1). CP increased significantly from pre to post exercise in both groups at baseline and in the placebo group after 14 weeks of treatment (p = 0.006). After 14 weeks, CP concentrations were tendentially lower with probiotics (p = 0.061). TOS was slightly increased above normal in both groups, at baseline and after 14 weeks of treatment. There was no effect of supplementation or exercise on TOS. At baseline, both groups showed considerably higher TNF-α concentrations than normal. After 14 weeks TNF-α was tendentially lower in the supplemented group (p = 0.054). IL-6 increased significantly from pre to post exercise in both groups (p = 0.001), but supplementation had no effect. MDA was not influenced, neither by supplementation nor by exercise.

**Conclusions:**

The probiotic treatment decreased Zonulin in feces, a marker indicating enhanced gut permeability. Moreover, probiotic supplementation beneficially affected TNF-α and exercise induced protein oxidation. These results demonstrate promising benefits for probiotic use in trained men.

**Clinical trial registry:**

http://www.clinicaltrials.gov, identifier: NCT01474629

## Background

Probiotic bacteria are described as live microorganisms that beneficially modulate microbiota and health of the host [1]. In the last few years they became increasingly popular as nutritional supplements especially to achieve reduction of gastrointestinal (GI) complaints and common infectious illnesses. In sports and exercise, there is some evidence for probiotics’ potential to reduce incidence and severity of respiratory tract infections [2,3], and to shorten the duration of GI symptoms in trained athletes [4]. Other studies report attenuation of exercise-induced increase in pro- and antiinflammatory cytokines after 11 weeks [5] and increased plasma antioxidant levels after 4 weeks of probiotic supplementation [6].

In performance sports there is a high prevalence of GI complaints among endurance athletes like runners and triathletes [7]. These problems are attributed to changed blood flow, that is shunted from the viscera to skeletal muscle or the heart [8]. Such exercise-induced reductions in intestinal blood flow as well as exercise-linked thermal damage to the intestinal mucosa can cause intestinal barrier disruption, followed by an inflammatory response [9]. Symptoms described are nausea, stomach and intestinal cramps, vomiting and diarrhea. The increased permeability of the instesinal wall leads to endotoxemia, and results in increased susceptibility to infectious- and autoimmune diseases, due to absorption of pathogens/toxins into tissue and blood stream [10-12]. Thus, to reduce exercise-induced GI permeability and its associated symptoms and illnesses, nutritional solutions like probiotic supplementation may be of relevance for athletes and also a real challenge for the probiotic industry to develop bioeffective products.

Tight junctions are protein structures that represent the major barrier within the intestinal paracellular pathway. They seal the paracellular space between epithelial cells and regulate the movement of fluid, macromolecules and leukocytes between the bloodstream and the intestinal lumen, and vice versa [13]. These complex structures consist of more than 50 proteins and are regarded to be key factors of GI permeability [14].

Commensal and probiotic strains modulate the amount of tight junction proteins at the cell boundaries and can prevent or reverse adverse effects of pathogens. Several probiotic strains such as *Lactobacillus plantarum*[15-17], *Bacteroides thetaiotaomicron* ATCC29184 [18], *Escherichia coli* Nissle 1917 [19], *Bifidobacterium longum* SP 07/3 and *Lactobacillus rhamnosus* GG [20] revealed beneficial impacts on tight junction- and intestinal barrier function. Moreover, various dietary components like polyphenols, proteins or amino acids are postulated to regulate epithelial permeability by modifying expression and localization of tight junction proteins in the paracellular space [14].

Zonulin - a protein of the haptoglobin family released from liver and intestinal epithelial cells - is described as the main physiological modulator of intercellular tight junctions so far. Increased zonulin concentrations are related to changes in tight junction competency and increased GI permeability [21]. The “leak” in the paracellular absorption route enables antigens to pass from the intestinal milieau, challenging the immune system to produce an immune response and subsequent inflammation and oxidative stress [13,22,23].

The effects of probiotics on GI barrier integrity, inflammation and oxidative stress, are not elucidated particularly in the context of sports and exercise. Hence, we focussed the primary outcome of this study to explore the effects of a multi-species probiotic supplement on GI permeability in endurance trained men. The secondary outcome of this trial was to evaluate whether the probiotic supplementation affects markers of oxidation and inflammation in plasma, before and after intense exercise.

## Methods

### Subjects

23 endurance trained men (triathletes, runners, cyclists) participated in this trial. Inclusion criteria: male, healthy, 30–45 years, non-smokers, trained (maximum oxygen uptake, VO_2max_ > 45 mL ^**.**^ kg^-1^^**.**^ min^-1^), no dietary or nutritional supplement use within four weeks prior to the first exercise test. Exclusion criteria: smokers, men who failed eligibility testing for exercise - as described by the Austrian and German standards in sports medicine [24], men who significantly changed training regimen during the study, chronic or excessive alcohol consumption, recent surgery or illness, body fat > 20%. Body fat content and distribution was estimated by a computerized optical device Lipometer (Möller Messtechnik, Graz, Austria), as described by Möller, et al. [25]. Besides inclusion and exclusion criteria, a standard blood chemistry panel was determined after an overnight fast and all subjects completed a medical history. Subjects characteristics are presented in Table 1.

**Table 1:** **Baseline characteristics, performance data, clinical chemistry and nutrition data of 23 trained men**^**1**^

**Variable**	**Reference range**^**2,3**^	**Probiotics (n = 11)**	**Placebo (n = 12)**
Age, yr		37.6 ± 4.7	38.2 ± 4.4
BMI, kg ^**.**^ m^-2^		23.7 ± 2.2	23.9 ± 3.1
Weight, kg		80.2 ± 7.9	81.6 ± 6.3
Total body fat, %		14.2 ± 3.1	14.4 ± 3.5
VO_2max_, mL		4118 ± 172	4087 ± 169
VO_2max_, mL ^**.**^ kg^-1 **.**^ min^-1^		51.2 ± 4.1	50.3 ± 3.6
P_max_, W		367 ± 28	357 ± 32
P_rel_, W ^**.**^ kg^-1^		4.53 ± 0.55	4.38 ± 0.62
Clinical Chemistry:			
Glucose, mmol ^**.**^ L^-1^	3.9–6.1	4.5 ± 0.5	4.7 ± 0.4
Hemoglobin, g ^**.**^ L^-1^	136–172	153 ± 12	155 ± 19
Iron, μmol ^**.**^ L^-1^	14–32	20.4 ± 4.5	18.6 ± 3.9
Ferritin, μg ^**.**^ L^-1^	18–300	101 ± 42	89 ± 36
Cholesterol, mmol ^**.**^ L^-1^	< 5.85	4.47 ± 1.23	4.56 ± 1.13
HDL, mmol ^**.**^ L^-1^	0.80–1.80	1.30 ± 0.13	1.33 ± 0.19
Triglycerides, mmol ^**.**^ L^-1^	< 1.80	0.87 ± 0.32	0.81 ± 0.36
Vitamin D_3_, nmol ^**.**^ L^-1^	75–250	98 ± 26	106 ± 31
Testosterone, nmol ^**.**^ L^-1^	10–31	16.3 ± 4.9	18.2 ± 4.1
Creatinine, μmol ^**.**^ L^-1^	50–110	87 ± 13	93 ± 19
Diet (exerpts):			
Energy, kJ ^**.**^ d^-1^	11776–13902	11989 ± 1803	12356 ± 2455
Fat, %	< 30% of kJ **•** d^-1^	34.5 ± 6.2%	35.9 ± 5.1%
Protein, %	10–15% of kJ **•** d^-1^	14.7 ± 2.1%	15.8 ± 3.2%
Carbohydrates, %	> 50% of kJ **•** d^-1^	47.9 ± 9.1%	46.5 ± 10.3%
Alcohol, %	< 3.5%	1.9 ± 1.2%	1.5 ± 0.9%
Water, mL	2600	3162 ± 595	3022 ± 952
Fibres, g	30	23 ± 7	21 ± 6
Vitamin C, mg	72–106	113 ± 58	118 ± 66
Vitamin E, mg	14	12 ± 5	15 ± 9
ß-Carotene, mg	4	3.1 ± 2.5	3.2 ± 2.7
Folate, μg	434–505	281 ± 155	244 ± 165
Vitamin B-6, mg	3.2–3.8	5.3 ± 2.9	5.1 ± 2.8
Vitamin B-12, μg	3.3–3.7	5.0 ± 2.8	5.8 ± 1.4
Sodium, mg	> 646	2610 ± 685	2446 ± 770
Potassium, mg	2171–2523	3162 ± 904	3406 ± 1251
Magnesium, mg	185–361	412 ± 134	444 ± 119
Calcium, mg	1085–1261	1108 ± 395	998 ± 327
Iron, mg	10.9–12.5	13.3 ± 4.6	14.5 ± 6.2

^1^Values are means ± SD, and did not differ between the groups (P > 0.05, Student’s t-test); ^2^Reference range for clinical chemistry parameters [26]; ^3^Reference values for dietary intake (RDA) in Germany, Austria, Switzerland [27], ranges presented here apply to physical active people; VO_2max_ = maximum oxygen uptake, P_max_ = maximum performance, Prel = Performance related to body weight.

### Ethical aspects, recruitment and randomization

All subjects provided written informed consent prior to participating in this investigation. This study was conducted according to the guidelines of the Declaration of Helsinki for Research on Human Subjects 1989 and was approved by the Ethical Review Committee of the Medical University of Graz, Austria. The trial was registered under http://www.clinicaltrials.gov, identifier: NCT01474629.

The study focused trained men and was advertised in the largest sports magazine of Austria. After a telephone screening conducted by the research team, 29 men volunteered for eligibility testing. From those, 24 men were eligible and entered the study program.

Subjects were randomized into blocks of six and sequentially numbered. To guarantee a balanced VO_2max_ distribution between groups (probiotics versus placebo) we conducted stratification via VO_2max_ rank statistics. Randomization code was held by a third party (Union of Sport and Exercise Scientists Austria) and handed over for statistical analyses after collection of all data.

### Study design and time schedule

This was a randomized, placebo controlled, double-blinded study. All eligibility testing (blood panel, eligibility for exercise, clinic check-up, medical history questionaire, one-on-one interview) was finalized at least four weeks prior to the first exercise test. At the morning of the first exercise test a standardized breakfast (3 hours prior to exercise) was provided. After the test, the investigator dispensed the randomized sachet supply according to the man’s VO_2max_-ranking. After 14 weeks taking the powder from sachets as directed, they returned their remaining sachets and the same test procedure was repeated. All subjects were checked by the physician before each exercise test.

### Dietary and lifestyle assessment

Subjects were instructed to maintain their habitual diet, lifestyle and training regimen during the fourteen weeks study and to duplicate their diet before each exercise testing/blood collection appointment as described below. Before the first triple step test, men completed a 7-day food record for nutrient intake assessment. Subjects subsequently received copies of their 7-day diet records and were instructed to replicate the diet prior to the second exercise tests. The breakfast before each exercise test was standardized for the entire cohort to limit nutrient variation due to self-selection on the morning scheduled for blood draws. The composition of this standardized breakfast 3 hours prior to the strenuous exercise tests is shown in Table 2. Diet records were analyzed for total calories, protein, carbohydrate, fat, cholesterol, fiber, water, alcohol, and several vitamins, minerals, and fatty acids using “opti diet” software 5.0 (GOEmbH, Linden, Germany).

**Table 2:** Composition of the standardized breakfast 3 hours prior to the strenuous triple step test ergometry

**Food**	**kJ**	**Protein (g)**	**Fat (g)**	**Carbohydrates (g)**
Coffee with milk (low fat) or Tea with lemon and honey (10g)	180	0-2	0-2	4-10
3 slices wheat or rye bread	1390	8	1	75
Butter 20 g	652	-	16	-
Marmalade/jam 30 g	343	-	-	19
One slice low fat ham	331	6	6	-
One piece of cheese	490	16	5	-
250 mL fruit juice	836	2	-	46
250 mL water	-	-	-	-
Total	4222	32-34	28-30	144-150
Meal energy %		13%	27%	60%

### Treatment

The men randomized to probiotics (n = 11) received boxes with sachets containing multi-species probiotics (Ecologic®Performance, produced by Winclove b.v., Amsterdam, the Netherlands; the product is also branded as OMNi-BiOTiC®POWER). The probiotic supplement contained of a matrix and six probiotic strains: *Bifidobacterium bifidum* W23, *Bifidobacterium lactis* W51, *Enterococcus faecium* W54, *Lactobacillus acidophilus* W22, *Lactobacillus brevis* W63, and *Lactococcus lactis* W58. The matrix consisted of cornstarch, maltodextrin, vegetable protein, MgSO_4_, MnSO_4_ and KCl. The placebo consisted of the matrix only. The minimum concentration was 2.5 × 10^9^ colony forming units (CFU) per gram. Subjects were instructed to take 2 sachets a 2 g per day (4 g/day), equivalent to 10^10^ CFU/day, with 100–125 mL of plain water per sachet, one hour prior to meals and throughout 14 weeks. Those subjects randomized to placebo (n = 12) received identical boxes and sachets with the same instructions for intake.

### Exercise tests

Each subject was instructed not to perform physical training 3 days prior to any exercise test.

For eligibility testing all subjects performed an incremental cycle ergometer exercise test (EC 3000, Custo med GmbH, Ottobrunn, Germany) at 80 rpm. After a three minute rest phase sitting inactive on the ergometer, work rate started at 60 W for three minutes and was increased 20 W every minute until voluntary exhaustion. This allowed subjects to reach exhaustion within 15–18 minutes. A standard electrocardiogram was recorded during the entire test, which was supervised by a physician. Respiratory gas exchange variables were measured throughout the incremental exercise tests using a breath-by-breath mode (Metalyzer 3B, Cortex Biophysik GmbH, Leipzig, Germany). During these tests, subjects breathed through a facemask. Oxygen uptake (VO_2_), carbon dioxide output (VCO_2_), minute ventilation (V_E_), breathing rate (BR) and tidal volume (V_T_) were continuously obtained. Heart rate (HR) was monitored throughout the tests using a commercially available heart rate monitor (Polar Vantage NV, Polar Electro Finland).

For the triple step test ergometry we used the same test protocol as described above but repeated the incremental exercise test for two times, so that three step tests until voluntary exhaustion were integrated in one single bout of exercise. After the first and second part of this triple test subjects performed for 15 minutes with 60 W at 80 rpm. After the third part subjects continued exercise for three minutes with 60 W and 80 rmp and stopped then. The whole test procedure lasted between 80 and 90 minutes, depending on duration of each step test/part.

Blood pressure was controlled after each 100 W and after the last step of each ergometry. Gas exchange variables were monitored continuously throughout the step tests as described above. During the 15 minutes intervals between the ergometry step tests the facemask was removed to consume 750 mL of plain water, in total over the whole test procedure.

Fourteen weeks later this procedure was repeated on the same cycle ergometer, with the same investigator, standardized room temperature (20°C) and humidity (60%).

### Blood and feces collection

We conducted blood collections in supine position from a medial cubital vein at each triple ergometry test: before exercise (Pre) and within 10 min post exercise (Post). Venous blood was collected to determine carbonyl proteins (CP), malondialdehyde (MDA), total oxidation status of lipids (TOS), tumor necrosis factor-alpha (TNF-α), and interleukin-6 (IL-6). After centrifugation for 10 minutes plasma was removed and samples were frozen at −70°C until analysis.

For zonulin and α1-antitrypsin from feces the subjects collected samples at baseline and after 14 weeks with standardized stool tubules within 24 hours prior to bringing the sample in a cool bag to the laboratory. All samples were analyzed within 72 hours after dispensing. Throughout the 14 weeks treatment the subjects recorded a stool protocol to monitor stool appearance with help of the Bristol stool scale/chart [28].

### Stool analyses

Zonulin and α1-antitrypsin were analyzed with commercially available ELISA kits (Immundiagnostik AG, Bensheim, Germany).

The zonulin analysis is based on a competition between the free antigen in the samples or standards and the antigen coated on the wells of the microplate. Standards, samples and the primary anti-zonulin antibody are transferred directly into the precoated microplate wells. The antigen in the samples competes with the antigen immobilized on the wells of the microplate for the binding sites of the specific anti-zonulin antibody. A peroxidase-conjugated antibody is used for detection, and tetramethylbenzidine as a peroxidase substrate. The enzymatic reaction is terminated by acidic stop solution. The quantification is based on the optical density at 450 nm. Data are expressed in ng/mL.

The assay for analyses of α1-antitrypsin utilizes the “sandwich” technique with two selected polyclonal antibodies that bind to the glycoprotein forming a “sandwich” of capture antibody - human α1-antitrypsin - peroxidase-conjugate. Tetramethylbenzidine is used as peroxidase substrate. Finally, an acidic stop solution is added to terminate the reaction. The colour changes from blue to yellow. The intensity of the yellow colour is directly proportional to the concentration of α1-antitrypsin. Samples are quantified by referring their optical density to a lot-dependant master calibration curve and the use of a calibrator that is run with each test. Data are expressed in mg/dL.

### Analyses of blood parameters

CP was analyzed with a commercially available ELISA (Immundiagnostik AG, Bensheim, Germany) via reaction of protein with dinitrophenylhydrazine (DNPH). The non-protein constituents and unconjugated DNPH are separated by ultracentrifugation. The proteins are adsorbed to an ELISA plate and incubated with anti-DNPH antibody followed by antibody-linked horseradish peroxidase. Absorbances are related to a standard curve prepared with oxidized serum albumin. The carbonyl protein content is calculated from the estimated carbonyl concentration and the total protein content of the sample. For this reason, a parallel determination of the protein content is required. Data are expressed in pmol/mg.

MDA was determined according to a previously described HPLC method by Pilz et al. [29] after derivatization with 2,4-DNPH. This method determines the protein bound MDA. The HPLC separations were performed with an L-2200 autosampler, a L-2130 HTA pump and a L-2450 diode array detector (all: VWR Hitachi Vienna; Austria). Detector signals (absorbance at 310 nm) were recorded and program EZchrom Elite (VWR) was used for data requisition and analysis. Data are expressed in nmol/mL.

Analysis of TOS: This assay (Immundiagnostik AG, Bensheim, Germany) determines total lipid peroxides and is performed by the reaction of a peroxidase with the peroxides in the sample followed by the conversion of tetramethylbenzidine to a colored product. After addition of a stop solution the samples are measured at 450 nm in a microtiter plate reader. The quantification is performed by the delivered calibrator. Data are expressed in μmol/L H_2_O_2_.

TNF-α was analyzed with a commercially available ELISA (Immundiagnostik AG, Bensheim, Germany) allowed quantitative determination of Tumor Necrosis Factor-α by using monoclonal antibodies and a horseradish peroxidase labeled conjugate. The amount of the converted substrate by the peroxidase is directly proportional to the amount of bound TNF-α and can be determined photometrically. Data are expressed in pg/mL.

IL-6 was also measured with commercial available ELISA kits (Invitrogen, LifeTech Austria, Vienna, Austria) using monoclonal antibodies specific for human IL-6. Based on the binding of streptavidin-peroxidase to antibodies the intensity of a colored adduct is directly proportional to the concentration of the cytokine and can be determined photometrically. Data are expressed in pg/mL.

### Blood chemistry panel

Standard blood chemistry values were determined after overnight fast using EDTA plasma from peripheral venous blood. Analyses were conducted with a routine clinical chemistry analyzer (Abbott Diagnostics, Vienna, Austria).

### Statistical analyses and sample size calculation

Per protocol analyses were performed using SPSS for Windows software, version 19.0. Data are presented as mean ± SD. Data for pre - post comparisons were adjusted for plasma volume changes as described elsewhere (except for CP, as it is expressed on protein) [30]. Statistical significance was set at P < 0.05. The Shapiro-Wilk test was used to determine normal distribution. Baseline characteristics, performance data, nutrient and clinical chemistry data, were compared by unpaired Student’s t-test. Data obtained for CP, MDA, TOS, TNF-α, and IL-6, were analyzed using a univariate, three-factorial, repeated measures ANOVA. Factors: treatment (probiotic supplementation and placebo), exercise (pre and post exercise), session (triple step test ergometry 1 and triple step test ergometry 2). For zonulin and α1-antitrypsin we used a two-factorial ANOVA (treatment, time). Significant interactions and main effects were analyzed by using Bonferroni correction.

Sample size calculation was based on oxidation markers CP and MDA. We estimated between 7 and 9 subjects per group - depending on parameter, standard deviation and effect size - to reach a probability of error (alpha/2) of 5% and 80% power. Allowing for a drop-out rate of 30%, 12 subjects per group were recruited.

## Results

### Study population and nutrition

**Figure 1:**
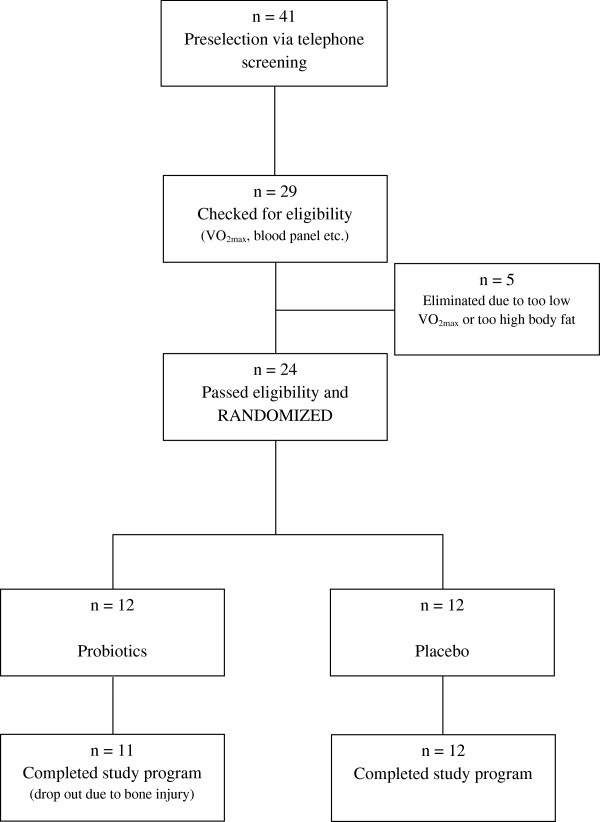
CONSORT diagram.

A CONSORT diagram outlining participant recruitment is depicted Figure 1. Of the 24 randomized men, 23 completed the full program and entered statistical analyses. There was one early termination in the probiotic group (n = 11). The man dropped out due to bone injury unrelated to the study.

Returned sachets count after the treatment period revealed a compliance >90% in both groups. Groups did not differ in age, BMI, body weight and fat, clinical blood chemistry variables, and diet (P > 0.05).

### Triple cycle step test ergometry

Performance data for VO_2max_, VO_2max_ related to body weight (relVO_2max_), maximum performance and performance related to body weight (P_rel_) are shown in Table 1. There were no significant differences between probiotic supplementation and placebo for these parameters (P > 0.05).

### Zonulin

**Figure 2:**
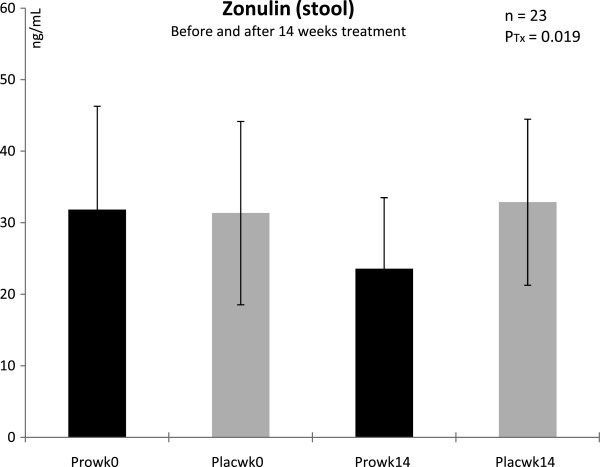
**Stool concentrations of zonulin in trained men before and after 14 weeks of treatment.*** Pro* with probiotics supplemented group, *Plac* placebo group, *Tx* treatment, *wk* week; n = 11 (probiotic supplementation), n = 12 (placebo). Values are means ± SD. There was a signficant difference between groups after 14 wk of treatment: P_Tx_ < 0.05 (ANOVA).

As zonulin was determined from feces we can only provide values from the last stool prior to exercise. The mean concentrations of zonulin were at baseline slightly above normal in both groups (ref. range: < 30 ng ^**.**^ mL^-1^, Figure 2). After 14 weeks supplementation with the multi-species probiotic supplement zonulin decreased into a normal physiological range and was significantly lower in the probiotic group compared to placebo (P = 0.019), this was corresponding to a decrease > 20%.

### α1-antitrypsin

There were no differences between groups at any time point assessed, neither with treatment nor with exercise. α1-antitrypsin concentrations in feces were within normal range at baseline and after 14 weeks of treatment (< 27.5 mg ^**.**^ dL^-1^, data not shown).

### Carbonyl groups on proteins, CP

**Figure 3:**
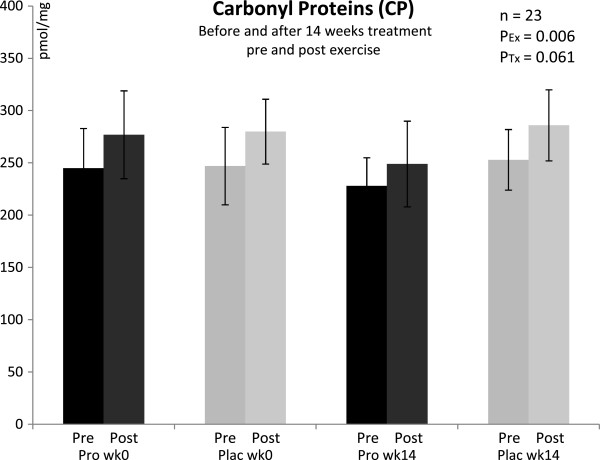
**Plasma concentrations of carbonyl groups bounded on protein in trained men before and after 14 weeks of treatment, and pre/post a triple step test cycle ergometry.*** Pro* with probiotics supplemented group, *Plac* placebo group, *Ex* exercise, *Tx* treatment, *wk* week; n = 11 (probiotic supplementation), n = 12 (placebo). Values are means ± SD. There was a significant differences from pre to post exercise (except “Pro wk14”): P_Ex_ < 0.05; and a tendential difference between groups after 14 wk of treatment: P_Tx_ < 0.1 (ANOVA).

Pre-exercise concentrations of both groups were 15–25% above normal (reference range < 200 pmol ^**.**^ mg^-1^). There were no differences between groups at baseline. The post-exercise increase was significant (P = 0.006). Post-hoc analysis revealed that this exercise-induced increase did not reach significance after 14 weeks of probiotic treatment. After 14 weeks, the supplemented group showed decreased CP concentrations pre and post exercise compared to placebo, but likewise this effect did not reach significance (P = 0.061) (Figure 3).

### Malondialdehyde, MDA

There were no differences between groups at any time point assessed, neither with treatment nor with exercise. MDA concentrations were unremarkable and within normal range (2.16 ± 0.39 nmol ^**.**^ mL^-1^, data not shown).

### Total oxidation status, TOS

The measured TOS values were above normal at all time points (reference range < 350 μmol ^**.**^ L_H2O2_^-1^). As with MDA, there were no differences between groups at any time point assessed, neither with treatment nor with exercise (data not shown).

### Tumor necrosis factor alpha, TNF-α

**Figure 4:**
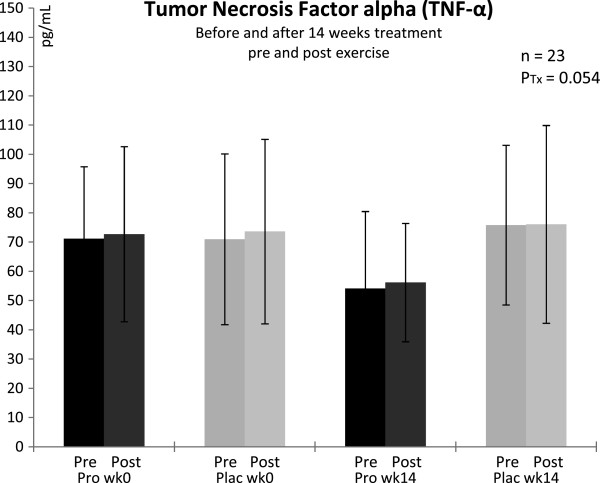
**Plasma concentrations of tumor necrosis factor-alpha in trained men before and after 14 weeks of treatment, and pre/post a triple step test cycle ergometry.*** Pro* with probiotics supplemented group, *Plac* placebo group, *Tx* treatment, *wk* week; n = 11 (probiotic supplementation), n = 12 (placebo). Values are means ± SD. There was a tendential difference between groups after 14 wk of treatment: P_Tx_ < 0.1 (ANOVA).

Despite the typical high standard deviations for TNF-α, due to well known cytokine inter-individual variability, the data were normally distributed. Concentrations at all time points were distinctly higher than normal (reference range < 20 pg ^**.**^ mL^-1^) with mean values > 50 pg ^**.**^ mL^-1^ (Figure 4). After 14 weeks of probiotic supplementation, TNF-α showed reduced concentrations compared to the placebo group but this effect barely failed significance (P = 0.054). Exercise had no effect on TNF-α.

### Interleukin 6, IL-6

**Figure 5:**
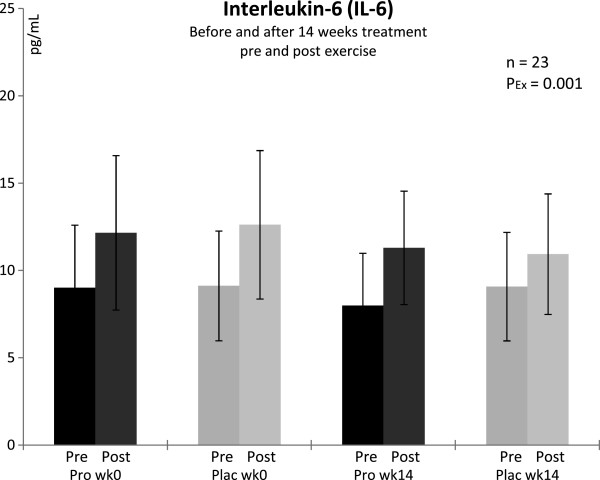
**Plasma concentrations of interleukin-6 in trained men before and after 14 weeks of treatment, and pre/post a triple step test cycle ergometry.*** Pro* with probiotics supplemented group, *Plac* placebo group, *Ex* exercise, *wk* week; n = 11 (probiotic supplementation), n = 12 (placebo). Values are means ± SD. There were significant differences from pre to post exercise: P_Ex_ < 0.05 (ANOVA).

There were no differences between groups at baseline and after treatment. IL-6 concentrations were unremarkable and within normal range before exercise (< 11.3 pg ^**.**^ mL^-1^), but we observed a significant increase from pre to post exercise above normal in both groups (P = 0.001, Figure 5) at baseline and after 14 weeks of treatment.

## Discussion

Athletes exposed to high intense exercise show increased occurence of GI symptoms like cramps, diarrhea, bloating, nausea, and bleeding [31,32]. These symptoms have been associated with alterations in intestinal permeability and decreased barrier function [33,34] and subsequent with inflammation and oxidative stress [22,23].

For this investigation we assembled a panel of surrogate markers related to increased intestinal permeability, oxidative stress and inflammation. The study was primarily focussed on the effects of 14 weeks multi-species probiotic supplementation on intestinal barrier function in trained men compared to a placebo group (primary outcome). The secondary outcome was to evaluate the influence of the probiotic supplementation and the model of exercise on markers of oxidative stress and inflammation. The resulting data show that, after the 14 weeks study period i) the probiotics decreased stool zonulin concentrations - a modulator of intestinal barrier function - from slightly above normal into the physiolgical range; ii) the probiotic supplementation decreased protein oxidation and the chronic inflammatory marker TNF-α; and iii) the model of exercise did not induce oxidative stress but increased concentrations of the inflammatory cytokine IL-6 in this cohort of endurance trained men.

### Markers of intestinal permeability

Zonulin is regarded as a phyiological modulator of intercellular tight junctions and a surrogate marker of impaired gut barrier [19,35-37]. Beside liver cells, intestinal cells can synthesize zonulin and the zonulin system can be activated by dietary proteins (especially gliadin) or enteric bacteria [21,38]. We can exclude a dietary influence on the observed changes in zonulin concentrations as our subjects followed strictly all dietary instructions and did not change their diet during the study period. To our best knowledge this study reports for the first time that probiotic supplementation can reduce zonulin concentrations in feces of trained men. The observed reduction is all the more remarkable as mean concentrations were slightly above normal at baseline (ref. range: < 30 ng ^**.**^ mL^-1^) and decreased into normal after 14 weeks with probiotics. These data indicate that our trained cohort suffered already a mild increase in intestinal permeability at baseline, probably due to chronic exercise training.

It seems that the 14 weeks of probiotic supplementation could reduce zonulin concentrations and hence improve intestinal barrier integrity. A mechanistic explanation for an improved intestinal barrier function after probiotic treatment is provided by Karczewski et al. [17]: they postulate that certain lactic bacteria might activate the Toll-like receptor 2 (TLR2) signaling pathway. TLR2 is localized in the membranes of intestinal wall cells to communicate with metabolites and/or bricks from e.g. Gram-positive bacteria [39]. Activation of the TLR2 signaling pathway has been shown to enhance epithelial resistance in vitro [40]. We suggest that the supplemented probiotics surpassed bacteria that activate the zonulin system (e.g. Gram-negative bacteria), settled in the deep intestine, and could probably activate the TLR2 signaling pathway. This hypothesis about the settlement of the supplemented probiotic bacteria is in part strengthened by observations of Koning et al. [41] who showed that *Enterococcus faecium* W54 - one of our used strains - significantly increases in feces after 2 weeks of multi-species probiotic treatment. Their findings demonstrate that these bacteria can survive gastric transport and colonize the GI tract. Thus, our observation on the zonulin decrease after probiotic supplementation could be of high practical relevance for athletes under the perspective that an improved intestinal barrier reduces athlete’s susceptibility to endotoxaemia and associated cytokine production [42].

α1-antitrpysin in feces is another marker that displays GI barrier integrity and is widely used to estimate protein leakage into the instestinal tract [43,44]. In this study α1-antitrypsin values did not change after probiotic treatment. We believe that, although our subjects showed indices of a mild disturbance of intestinal permeability at baseline, this slight imbalance in intestinal barrier function was not distinctive enough to provoke an acute-phase response in liver cells via increased α1-antitrypsin synthesis.

### Oxidative stress markers

Protein oxidation can result in loss of enzyme and protein structur and function [45]. Reactive oxygen and nitrogen species, free metal ions and lipid oxidation end products can generate CP [46]. In this cohort, protein oxidation, as indicated by CP, was already increased at baseline in both groups. These data suggest a higher level of protein oxidation in this group performing permanent physical exercise training. The increased resting CP concentrations but also the post-exercise increase in trained men of this age are not really surprising. We already demonstrated that men under certain psycho-physical stress profile can show increased protein oxidation above normal at rest [47] and that high intense exercise also leads to increased CP [48]. The interesting and new observation in this study was that CP concentrations decreased by a trend with probiotics and that the post-exercise increase did not reach significance anymore after probiotic treatment. Although only a trend, we hypothesize that there could be a link between disturbed intestinal barrier, probiotic supplementation and protein oxidation. Some probiotic strains might exert antioxidant activities that could beneficially influence protein oxidation in plasma. Subsequent studies with a higher number of subjects might help to investigate a possible relation. It would be also interesting to observe if a longer time period or higher dosages of probiotic supplementation could lower CP values into a normal range (reference range < 200 pmol ^**.**^ mg^-1^).

MDA, a widely used marker to estimate lipid peroxidation [49-51], did not respond to probiotic supplementation. We measured bound MDA as an indicator of older damage on PUFA [51]. However, we observed no effect, indicating minor or no interaction of the nutraceutical with this group of fatty acids.

TOS represents the amount of total lipid peroxides. It is an all-over indicator of lipid peroxidation, and thus not as specific for oxidation on certain molecules like MDA. Values in both groups were above the reference range (< 350 μmol ^**.**^ L_H2O2_^-1^) at baseline and at the end of the study. As for CP, these data indicate a higher level of oxidation in this group under permanent physical exercise training. However, in contrast to CP, this surrogate marker was not influenced by the probiotic treatment.

### Markers of inflammation

TNF-α is a pro-inflammatory cytokine and a central mediator of systemic inflammatory response. Leucocytes, endothelium and adipocytes produce TNF-α but strenuous exercise has only limited impact on its release, compared to IL-6 [52]. This is also confirmed by our data that did not show an exercise-induced effect on TNF-α in both groups. Interestingly, our subjects showed significant increased values above normal (reference range < 20 pg ^**.**^ mL^-1^) at all measured time points. Probiotic supplementation reduced these high values about 20% but this reduction did neither reach the normal range nor significance (P = 0.054). However, our results let us hypothesize that the trained men suffered a state of chronic low-grade inflammation due to decreased intestinal barrier function which was likely evoked by chronic exercise stress. The data indicate that there is a potential for probiotic supplementation to reduce this systemic low-grade inflammation indirectly via improvement of gut barrier function.

In contrast to TNF-α, IL-6 is a cytokine which increases significantly in plasma with strenuous exercise as it originates primarily from the contracting sceletal muscles [52]. During exercise the production of IL-6 seems to be a TNF-independent pathway [53]. We also observed significantly increased IL-6 concentrations after the strenuous exercise tests. The probiotic supplementation had no influence on this IL-6 release. Obviously the exercise-induced, muscle-derived, increase in IL-6 is not related to intestinal barrier integrity. This suggestion is also supported by the normal IL-6 values at rest and basline - in contrast to TNF-α. These normal IL-6 values indicate that basic IL-6 production was not affected by chronic exercise training or by the observed mildly decreased gut barrier function.

### Limitations of the study

We observed only trends for decreased TNF-alpha (P = 0.054) and CP (P = 0.061) indicating that this study was slightly underpowered for some outcomes. As we did not find one study on zonulin and probiotic supplementation in trained men to orientate, our sample size calculation was based on CP and MDA and on our experience with enteral absorbed antioxidant concentrates [48,50]. Obviously our assumptions for sample size calculation cannot be drawn into account when probiotic supplements are used - at least with the study design chosen in this project. Post hoc analysis revealed that 13 subjects per group for TNF-alpha and 15 subjects per group for CP would have been enough to get significant results. However, future studies with similar design should consider a total sample size of at least 30 subjects or a longer time period of treatment.

Another limitation of the study was the small number of measured parameters. This study was primarily focused on the effects of probiotics on zonulin in trained men. Subsequent studies should include a wider panel of surrogate markers in stool and serum to raise options to identify rationales and mechanisms. Parameters like corticotropin- releasing hormone (CRH), indicating activation of mast cells that stimulate tight junctions, or ß-hexosaminidase, several growth factors, an extended range of cytokines as well as the assessment of different fecal bacteria should be included.

## Conclusions

In conclusion our data support the hypothesis that an adequate probiotic supplementation can improve intestinal barrier function, redox hemostasis and low-grade inflammation in men under sustained exercise stress. Subsequent studies that focus on leaky gut associated consequences like endotoxaemia, athlete’s susceptibility to inflammation, infections, and allergies will be of high practical relevance.

## Abbreviations

ANOVA: Analysis of variance; AU: Arbitrary units; BMI: Body mass index; BR: Breathing rate; CFU: Colony forming units; CONSORT: Consolidated standards of reporting trials; CP: Carbonyl groups bounded on protein; DNPH: Dinitrophenylhydrazine; Ex: Exercise; GI: Gastrointestinal; MDA: Malondialdehyde; Plac: Placebo; P_max_, P_rel_: Maximal performance and relative to body weight; Pro: With probiotics supplemented group; RONS: Reactive oxygen and nitrogen species; SD: Standard deviation; TLR: Toll-like receptor; TOS: Total oxidation status; Tx: Treatment; V_E_: Ventilation per minute; VO_2_: Oxygen uptake; VCO_2_: Carbon dioxide output; VO_2max_: Maximum oxygen uptake; V_T_: Tidal volume.

## Competing interests

This project has been awarded competitive research grants from Winclove b.v., Amsterdam, The Netherlands, and Institut Allergosan, Forschungs- und VertriebsGmbH, Graz, Austria to the Institute of Nutrient Research and Sport Nutrition to yield research data regarding the use of probiotics in sports and exercise.

## Authors contributions

ML: principal investigator, development of overall research plan/study protocol, project management and study oversight, statistical analyses, preparation of manuscript. SB: blood sampling, laboratory logistics, statistical analyses, manuscript revision. GS: supervising physician, blood sampling, manuscript revision. KS: performance diagnostics, manuscript revision. FF: 2nd supervising physician, blood sampling, manuscript revision. SH: laboratory analyses, preparation of manuscript. BS: laboratory analyses, data collection, manuscript revision. JG: laboratory analyses, manuscript revision. All authors read and approved the final manuscript.
